# Evaluating plans for sustainable development in Arctic cities

**DOI:** 10.1007/s13280-023-01974-6

**Published:** 2024-04-09

**Authors:** Benjamin DiNapoli, Matthew Jull

**Affiliations:** Department of Architecture, University of Virginia, 110 Bayly Dr, Charlottesville, VA 22903 USA

**Keywords:** Arctic, Comparative analysis, Indicators, ISO 37120, Sustainable development, Urban planning

## Abstract

Cities located in the Arctic often have extreme geographic and environmental contexts and unique sociopolitical and economic trajectories that, when combined with amplified effects of climate change in the region, impact future sustainable development. Well-recognized and standardized sustainable development indicator (SDI) frameworks such as ISO 37120 or UN-Habitat City Prosperity Index are often used to compare data across cities globally using comprehensive sets of indicators. While such indexes help characterize progress toward development and guide short- and long-term decision-making, they often lack relevance to specific contexts or characterize future visions of urban growth. To evaluate the extent of these deficiencies and to provide a comparative analysis of approaches to sustainable urban growth in the Arctic, this paper analyzes city planning documents for five northern cities - Anchorage (USA), Utqiagvik (USA), Reyjavik (ISL), Iqaluit, (CAN), Whitehorse, (CAN) - for goals, targets, and indicators and compare these to thematic areas and indicators defined by ISO 37120:2018 Sustainable Cities and Communities. The results confirm that although international SDI frameworks may be useful for comparative analysis of cities across diverse regions, they exclude important local factors that influence goal-oriented urban sustainability planning strategies employed in the Arctic region.

**Supplementary Information:**

The online version contains supplementary material available at 10.1007/s13280-023-01974-6.

## Introduction

The Arctic is experiencing amplified effects of climate change, as well as increased natural resource extraction, urbanization, and population growth (Ford et al. 2021). These changes are creating pressure on cities in the region to develop holistic models for sustainable development that consider factors such as housing, infrastructure, city services, and economic and business diversity. As city planning and policy practice have shown since the Brundtland Commission report, the current global approach to sustainable development is governed by four “pillars”: environmental management, social development, economic development, and urban governance (WCED 1987; National Research Council 2010; UNDESA 2013). These are the main drivers for policy-making and urban planning and incorporate principles of urban form, transportation and green infrastructure, renewable energy and waste management, social equity and environmental justice, economic development, health, and quality of life (Dizdaroglu 2017). At the city level, these core principles are implemented in the built environment through sustainability planning and land use frameworks which guide short- and long-term urban development by establishing ordinance and regulations and through visioning, goal and target setting, and annual reporting.

Within such frameworks, indicators are needed for cities to measure and monitor progress toward achieving those goals for sustainable development (Klopp and Petretta 2017; Nilsson and Larsen 2020). Indicators are increasingly used by municipal authorities to validate cities’ sustainable development strategies by enabling monitoring, assessment, and benchmarking (Tanguay et al. 2010). Indicators are aggregated into indexes that can be used to identify current conditions, trends, and challenges for a city. Such indexes can also assist policy-makers in decision-making processes by providing a means to evaluate the progress of urban development and sustainable performance among cities of different sizes, classes, and contexts (ISO 2014a). However, these indexes are often developed using a top-down approach and are focused on identifying indicators that are most useful for communicating the results in ways that inform policy-makers, the public, and others through outcome measures (e.g., energy usage, housing availability) (National Research Council 2010).

In response to growing interest for the global comparison of urban sustainability, the International Organization for Standardization (ISO) developed *ISO 37120 Sustainable cities and communities — Indicators for city services and quality of life* (ISO 37120)*,* a comprehensive set of indicators that measure the performance of municipal services and quality of life that are meant to apply to cities globally, “irrespective of size and location or level of development” (ISO 2014b). This paper examines how cities in the Arctic region can be characterized in terms of sustainable performance when using an international SDI framework such as ISO 37120. Building on a much larger study of urban sustainability in the Arctic (NSF PIRE, Award #1545913), the comparative analysis presented in this paper reviews and evaluates publicly available plans for sustainable development for a subset of five Arctic cities (Anchorage, AK; Utqiaġvik, AK; Whitehorse, CA; Iqaluit, CA; Reykjavik, IS), including sets of indicators developed by those cities to measure their performance, in relationship to the standardized indicators set forth in ISO 37120. In so doing, this paper seeks to better understand (1) the capacity of these five cities to develop sustainable development plans, (2) how the sustainable development plans of Arctic cities are structured in relation or in response to local-level urban, environmental, and social issues, (3) the ability of ISO 37120 to adequately characterize sustainable development planning goals, objectives, and targets that are specific to cities located in the Arctic region, and (4) areas of strengths or weaknesses of sustainability planning in Arctic cities in comparison to internationally accepted standards for sustainability reporting. The results of this analysis can be used to better develop holistic and integrated approaches to sustainability planning for Arctic cities in ways that aid the development of shared and coordinated approaches to visions of Arctic urban resilience.

## Materials and methods

### Sustainability indicators and the city

A wide range of sustainability indicators are used in practice for characterizing sustainable development in urban areas across the world. These indicators vary according to their needs, goals, and intended scale of application whether to measure the performance of a building, a city, a region, or greater (Brandon and Lombardi 2005; Shen et al 2011). For example, building-scale frameworks such as LEED, developed by the U.S. Green Building Council in 1993, BREEAM (United Kingdom in 1993) or BEPAC (Canada in 1994) evaluate environmental and ecological factors as they relate to buildings, their construction and operations, and quality of life of their occupants. City-scale frameworks such as the Sustainable Cities Index, developed in 2015 by the sustainable design consulting practice Arcadis, focus on the social, economic, and physical attributes urban settlements and their development (Arcadis 2022). At the global level, the United Nations (UN) 2030 Agenda for Sustainable Development and the associated Sustainable Development Goals (SDGs) were established in 2015 as a guiding international framework that provides the means—proposed actions and policies—for UN member states to achieve sustainable social, environmental, and economic development by the year 2030. In 2017, UN member states endorsed a global indicator framework across 244 indicators for measuring progressing on implementing the 17 SDGs and associated 169 targets. Recognizing the role of cities in achieving sustainable development, the SDG framework has also been “refined specifically for cities” by UN-Habitat through their New Urban Agenda (NUA), which expands on Goal 11 (Sustainable and Resilient Cities and Human Settlements) and focuses on human settlements and other urban-based targets across 85 indicators (UN-Habitat 2017).

Each type of framework described above can be used to evaluate changes over time and employ indicators that are relevant to their respective scale. However, one criticism of international SDI frameworks is that they lack relevance to any specific context and have difficulty characterizing future visions of the city in terms of sustainable development (Burford et al. 2013; Skold et al. 2018). For example, while both the SDG and NUA indicator frameworks are important and are intended to assist member states in “building out existing plans, supporting transitions to the SDG framework, and other national strategies” and sustainable development policies, both are necessarily broad to accommodate a diversity of approaches that depend on a country’s level of development, capacity, policies, or priorities (UN 2015). One recent study comparing the SDG and NUA indicator frameworks found that only 11 percent of SDG indicators and 40 percent of NUA indicators were relevant to measuring city planning and health outcomes, noting that a more comprehensive set of city-specific indicators would allow cities to better benchmark their progress toward sustainable development and allow for between-city comparisons (Giles-Corti et al. 2020). It has also been discussed that other SDI frameworks focus largely on providing indicators that are most useful for communicating with policy-makers at the expense of fully describing the sustainable profile of a city (Hajer et al. 2015). International SDI frameworks such as ISO 37120 help fill this gap because they are designed specifically to measure the performance of city service and quality of life at local, municipal, and subregional levels. But a recent study showed that fewer than half of ISO 37120 indicators measured issues of sustainability across 19 categories of core, profile, and supporting indicators; these indicators  instead provide measurements that describe a broad group of city service sectors that thematically apply to all cities equally across multiple world regions (Table 1; Berman and Orttung 2020).

**Table 1:** The thematic areas and total number of indicators included in ISO 37120:2018

ISO 37120 category	No. of indicators
Economy	11
Education	6
Energy	9
Environment and climate change	9
Finance	6
Governance	4
Health	6
Housing	10
Population and social conditions	9
Recreation	2
Safety	10
Solid waste	10
Sport and culture	3
Telecommunication	2
Transportation	9
Urban/local agriculture and food security	4
Urban planning	7
Wastewater	4
Water	7
Total: 19	128

When applied to Arctic cities—which are often located in remote contexts and cold climates, at high latitudes, with varying availabilities of resources, socioeconomic contexts, and indigenous populations—a key question arises of how to compare very different cities using indicators developed from a global perspective, thus lacking local-based measures of urban sustainability. Furthermore, at the onset, ISO 37120 does not reflect principles or contain indicators that measure whether a city has an inherent infrastructure in place to prepare plans for sustainable development or has the capacity to implement and monitor projects that result from such planning processes. This paper does not propose to create an SDI framework that only applies to one specific context but instead assesses whether an international SDI framework such as ISO 37120 can provide meaningful measurements of the results of long-term sustainable development planning in Arctic cities. It is first necessary to understand *how* Arctic cities structure their sustainable development plans, and *what* thematic areas, criteria, and indicators Arctic cities employ through such plans that result from local, regional, and climate-specific factors.

### Characterizing Arctic sustainable development plans

**Fig. 1:**
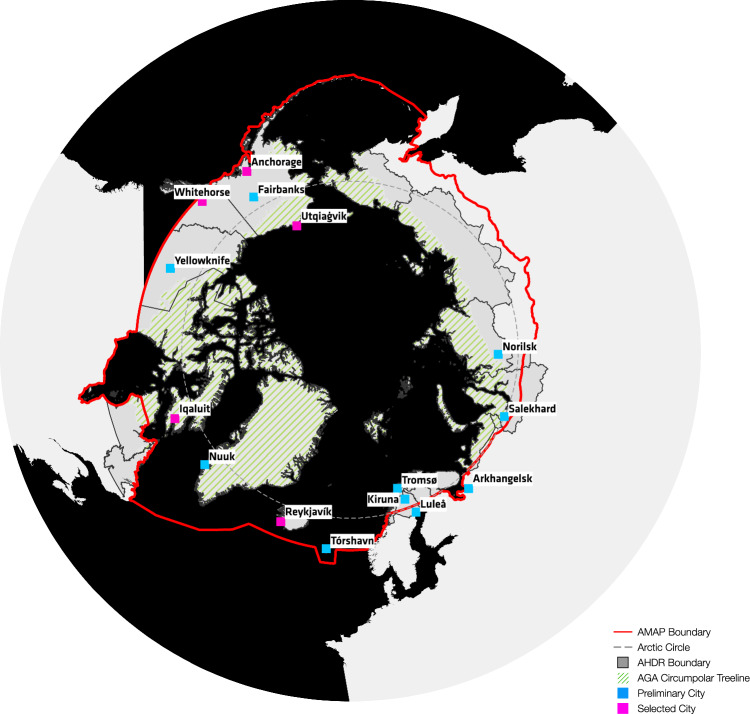
Circumpolar map of the initial set of Arctic cities used for comparative analysis

The set of cities included as part of the initial study *NSF PIRE: Promoting Urban Sustainability in the Arctic* (NSF Award #1545913) consisted of 46 settlements with populations over 12 000 people (Orttung et al. 2021). This selection of cities was based on a definition of the Arctic Region within an aggregated southerly boundary that includes the Arctic AHDR^1^ Boundary, AMAP^2^ assessment area, Arctic CAFF^3^ Boundary, and the Arctic Council EPPR^4^ boundary, as illustrated in Fig. 1. Of these cities, 15 were selected for further analysis for this study based on the availability and accessibility of sustainability planning documents (Table 2).

**Table 2:** Urban profiles of the initial set of 15 Arctic cities

Region	Country	No	City	Latitude	Population	Area (sq. km)	Source
North America	Canada	1	Iqaluit	63.7467°	7429	52.5	Statistics Canada (2021)
		2	Yellowknife	62.4545°	20 340	12.5	Statistics Canada (2021)
		3	Whitehorse	60.7209°	28 201	38.2	Statistics Canada (2021)
	United States	4	Utqiagvik (Barrow)	71.2906°	4927	55.6	Alaska Dept of Labor (2020)
		5	Fairbanks	64.8453°	31 515	82.1	Alaska Dept of Labor (2020)
		6	Anchorage	61.2175°	291 247	257.1	Census Bureau (2020)
Europe	Denmark	7	Tórshavn	62.0100°	14 053	7.8	Statistics Faroe Islands (2023)
	Greenland	8	Nuuk	64.1835°	19 604	30.4	StatBank Greenland (2023)
	Iceland	9	Reykjavík	64.1355°	141 010	95.3	Hagstofa Islands Statistics Office (2023)
	Norway	10	Tromsø	69.6510°	41 434	22.9	Statistics Norway (2023)
	Sweden	11	Kiruna	67.8555°	22 461	23.7	Statistics Sweden (2022)
		12	Luleå	65.5839°	47 956	42.9	Lulea Kommun (2023)
Russia	Russia	13	Salekhard	66.5305°	47 910	72.3	Russian Census (2021)
		14	Norilsk	69.3458°	174 453	18.7	Russian Census (2021)
		15	Archangelsk	64.5384°	301 199	257.4	Russian Census (2021)

**Fig. 2:**
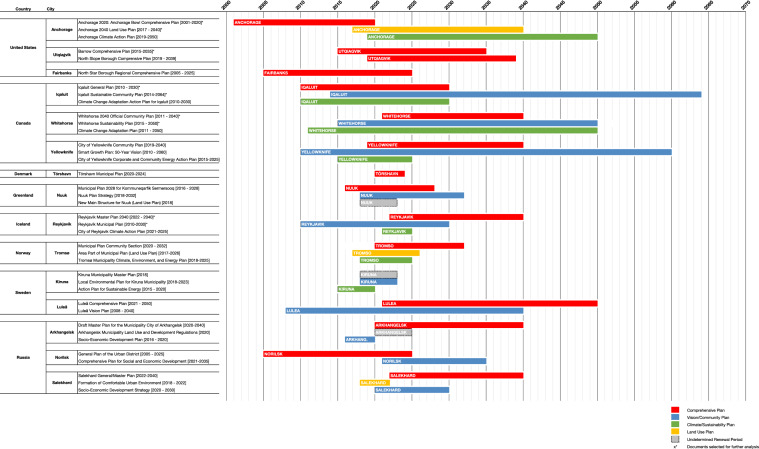
Scope, type, and timelines of primary sustainability planning documents

The primary set of planning documents for each city was based on (1) publicly available online documents using searches initiated with the name of the city and the keywords *comprehensive plan, climate change plan, vision, master plan, action plan, sustainability plan, land use plan,* and *strategy*, and (2) official municipal and regional websites for each city. Where available, plans were reviewed in English or translated from the document’s native language to English using Google Translate language processing services. The type of document (e.g., comprehensive plan, vision plan, land use plan, etc.), the publication date, and the revision period of the plan were recorded, if available. The results of this initial survey of long-term sustainability planning documents for each city are illustrated in Fig. 2

.

These documents provided a basis for evaluating the extent, quality, and frequency of sustainability planning across four categories: (1) the planning capacity for each city, including the type, number, and structure of municipal departments, the lifespan and revision period for planning documents, and the accessibility of such information to the public; (2) the ability for a city to integrate sustainable development principles into their guiding frameworks, including goal setting, multistakeholder coordination in planning processes, and plan benchmarking; (3) the extent of public participation and community input in planning processes; and (4) the ability for a city to implement capital projects resulting from sustainability planning through project goals, targets, monitoring, and financial strategies.

While there was variability among the plans reviewed in this survey that resulted from different planning practices, policies, and the availability of such documents to the public, it was evident that those plans with the longest revision periods—up to 50 years—tended to be community or sustainability plans. These plans focus on establishing broad sustainability goals, targets, and objectives for cities to meet using policies enacted in the comprehensive plan, which have shorter revision periods ranging from 5 to 25 years. Importantly, each of the 15 cities had a comprehensive plan. Two-thirds of the cities had vision or community plans; the exceptions included U.S cities, Tromsø, and Tórshavn. Just under one-half of the cities had a climate or sustainability plan, including all Canadian cities, and excluding all cities in Russia and most in the United States. Only one-third of the cities had a separate land use plan in addition to a comprehensive plan, notably most cities in Russia, Anchorage, Nuuk, and Tromsø. This survey also revealed that most plans are re-evaluated in shorter time periods than the overall lifespan of the document, which helps cities assess whether a plan adequately reflects changing values, goals, forecasts, and trends. In addition, most plans also state which document the current plan supplants, the date for which the current plan takes effect, and the anticipated end date of the plan.

Based on this preliminary review, the availability and completeness of planning documents, and to provide diversity of size, geography, and context between cities for the purpose of comparative study, five cities were selected for further analysis (Table S1). These include Anchorage, AK (USA); Whitehorse, YK (Canada); and Reykjavik, (Iceland). Two indigenous-governed cities that were not part of the initial set of 46 cities and have populations less than 12 000 were also included: Utqiaġvik (Barrow), AK (USA); and Iqaluit, NT (Canada). Each of the five selected cities has an important administrative and functional role within their respective territory. Anchorage is a major shipping port, tourism destination, and logistics thoroughfare to northern Alaska. Reykjavik as a primary hub for a growing tourism industry and for air transportation to Europe. Whitehorse is the largest city in Northern Canada and the capital of the Yukon. Utqiaġvik and Iqaluit, two of the northernmost and least populated cities in this study, have robust and long-term plans in place that prioritize community participation and the integration of cultural values in their planning processes. Both cities are also cooperatively managed by both municipal and indigenous councils. Furthermore, all five cities included in this study have a current set of planning documents that clearly outline goals, targets, and visions for future urban growth on both short- (within 5 to 10 years) and long-term (greater than 10 years) timeframes and contain indicator frameworks intended to measure progress toward achieving such benchmarks through annual reporting.

### Methods for comparison

City planning involves the ways in which land and its uses, resources, and services are planned, distributed, provided, and managed within a city. This can encompass various processes, ranging from detailed land use regulation (e.g., zoning) to broader, higher-level strategies and policies aimed at guiding social, economic, and environmental management and development. Within larger frameworks for sustainable development at the country or regional levels, in both urban and rural landscapes, city planning allows for sustainability goals to be achieved through sustainable development processes, policy implementation, and communication of those plans to the public, landowners, businesses, and other stakeholders. Globally, the methods for city planning are diverse and their organizations and structures depend on many factors, such as regional and national policy, the administrative role of a city within a territory, the authority of a municipality to regulate land uses, or even its size, population, or level of funding. In practice, urban and land use planning is coordinated through a municipal entity and set out through ordinance (e.g., policy that is enforceable by law), in addition to other non-binding reports, guidelines, plans, and strategies that outline visions for future urban development. While the organization of municipal planning varies for each of the five cities in this study, each has planning frameworks in place to establish policy, make recommendations, and receive funding from both state and national authorities.

This study reviewed publicly available primary planning documents that can be categorized as either *comprehensive plans* or *vision plans.*^5^ Comprehensive plans differ from vision plans in that they provide a legal framework for making decisions about urban development, land use, transportation, public facilities, economic development, housing, and other factors that are vital to a healthy and livable community to guide investment, establish regulation, and initiate capital projects (American Planning Association 2015). In contrast, vision plans are goal-driven and outcome-oriented plans often developed through engagement with community and local stakeholders. Vision plans identify sets of goals to be incorporated into or addressed by a city’s comprehensive plan, which help to establish a long-term foundation for future planning efforts (Tuiskunen et al. 2015). An important distinction between a comprehensive and vision plan is that the latter is, in most cases, non-binding, broad in scope, and tends to operate on longer timeframes with less frequent periods for re-evaluation and revision. For example, whereas both a vision and comprehensive plan may identify goals (e.g., general benchmarks for a community to reach in the future), a comprehensive plan will typically also identify policies corresponding to each goal (e.g., specific actions, statements of intent, plans, or ordinances), often in conjunction with land use planning and sets of strategies to help implement that policy.

In addition, many cities publish extensive secondary documents that can include both official and unofficial plans, reports, and guidelines (e.g., a community economic development strategy, or a waste management action plan). Such documents outline specific and coordinated goals and objectives for various sectors of city services to act on over the course of the plan period. In this study, it was found that secondary documents were often incorporated into the holistic frameworks of the primary plans or, conversely, were developed because of specific actions outlined in the primary plans. Furthermore, annual reporting (e.g., indicators) tended to reflect progress toward achieving those goals and targets set forth in a city’s primary planning documents. Therefore, the scope of the following analysis was limited to primary planning documents, with secondary documents incorporated into the analysis only if they (1) used clearly-defined indicators for annual reporting and (2) presented a coordinated, and holistic vision for future sustainable development.

Tables 3 and 4 describes the plan frameworks for both the primary vision and comprehensive plan for each city in this study, organized by thematic area. The total number of indicators, goals, or targets under each theme is provided. While Utqiaġvik is the only city in this study with a single primary plan (e.g., Barrow Comprehensive Plan 2015–2035), those goals that provide the basis for long-term sustainability planning and were used to define strategies to implement each objective were included for comparison to the other vision plan frameworks in Table 3. Notably, sets of indicators tended to be defined in the city’s vision plan, rather than the comprehensive plan, where they were often paired with those goals and targets used to define the long-term planning visions for each city.

**Table 3:**
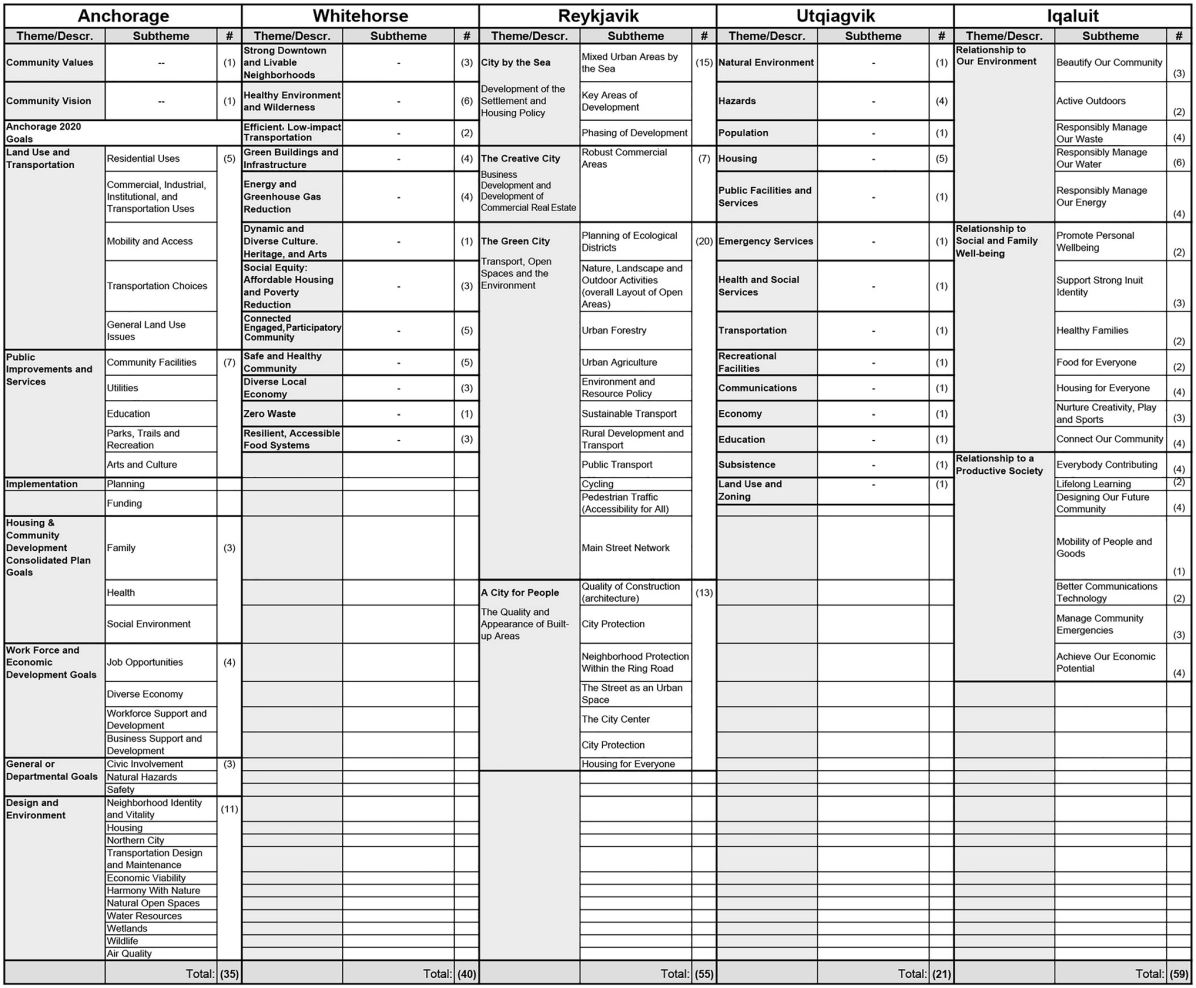
Comparison of the sustainability plan frameworks for five Arctic cities

**Table 4:**
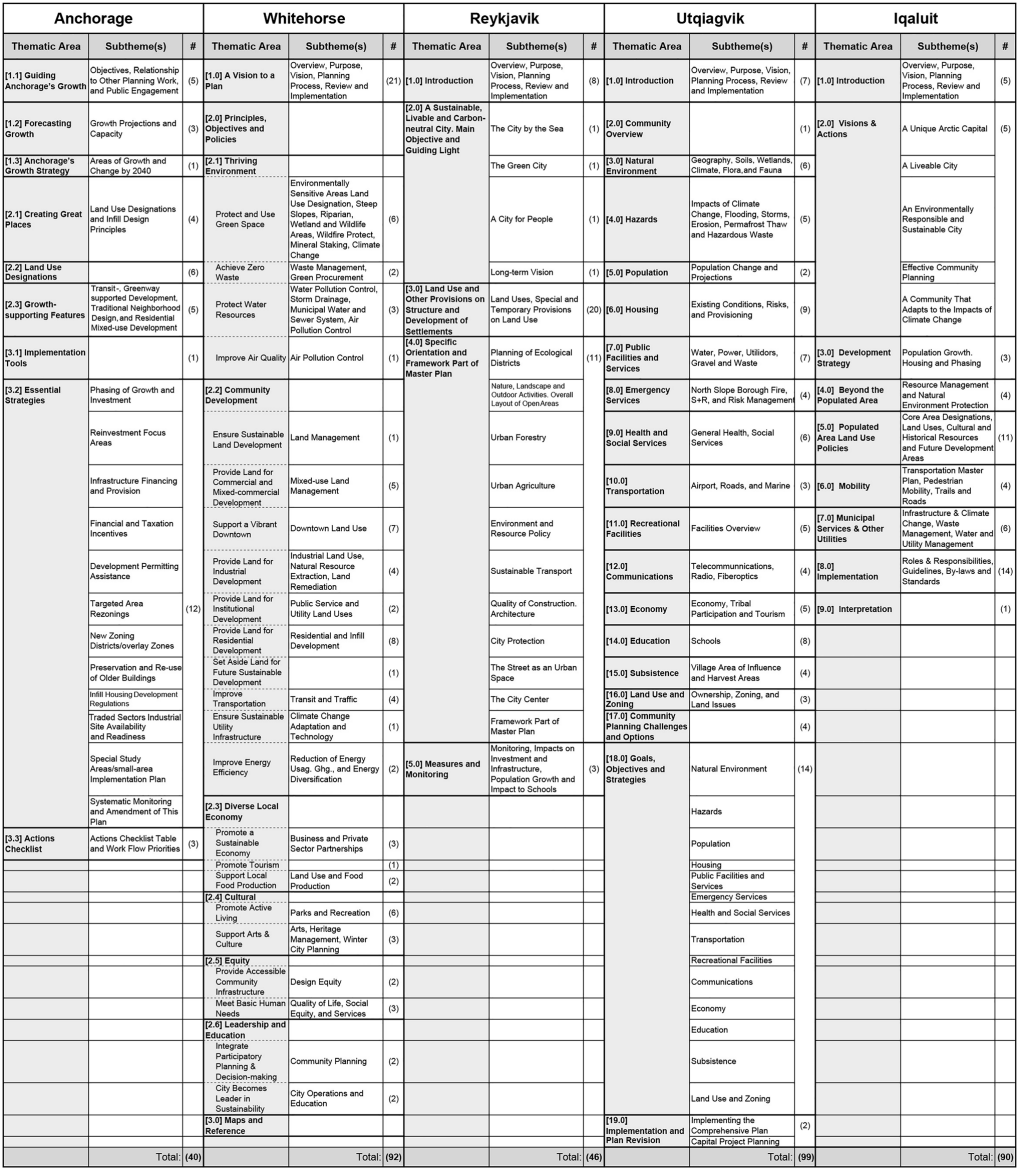
Comparison of the comprehensive plan frameworks for five Arctic cities

These documents were analyzed to identify the following: (1) thematic areas, (2) goals, targets, policies, and/or strategies within each thematic area, and (3) indicators used to measure progress toward achieving those goals. The thematic areas and indicators in ISO 37120 were then compared to the planning frameworks as defined above. A score ranging from 0 to 3 was assigned to each thematic area of the plan frameworks for each city, where (0) the plan thematic area does not contain any goals or indicators that correspond to the similar ISO 37120 thematic area; (1) the plan thematic area contains some goals or indicators but underperforms compared to ISO 37120; (2) the plan thematic area provides equivalent goals or indicators to ISO 37120; and (3) the plan thematic area provides more comprehensive goals or indicators than ISO 37120. This is a common method used for assessing the quality of plans across a range of planning domains (Stevens et al. 2014; Nilon et al. 2017). If no indicators were provided in a plan thematic area but either goals, targets, policies, and/or strategies were, those objectives were reviewed to determine if they sufficiently described or outlined the same intent as the relevant indicator in ISO 37120 and assigned a score using the methodology described above. In the context of this analysis, the term “performance” was used to describe the ability of the goals, targets, objectives, or indicators included in a thematic area of a plan framework to measure or monitor progress toward increasing the sustainability of a city. For example, an area that underperforms with respect to ISO 37120 presents less comprehensive, useful, or relevant metrics within that thematic area than those presented by ISO 37120. A score of “2” or greater indicates where a plan framework outperformed those metrics presented by ISO 37120. This method for comparison revealed what thematic areas of sustainable development Arctic cities prioritize and detail in their primary planning documents, whether those themes can be adequately measured and monitored using an international SDI framework such as ISO 37120, and the set of indicators used by each city to evaluate those themes.

## Results

A comparison between the 19 categories of indicators in ISO 37120 to the sustainability planning frameworks for the five cities is shown in Table 5. It is clear from this scorecard that Iqaluit, Anchorage, and Utqiaġvik have more comprehensive planning frameworks than the other cities, with almost all the thematic areas either meeting or exceeding those metrics presented by ISO 37120. Iqaluit has a score of 3 (e.g., presents more useful metrics than ISO 37120) across 10 of 19 categories, and a score of 2 (e.g., presents equivalent metrics to ISO 37120) across 8 categories—only underperforming for the thematic area of Finance, which is a shared attribute among all five cities. Anchorage has a score of 3 for 7 categories, including Economy, Energy, Governance, Urban Planning, and Health, among others. All five cities outperformed ISO 37120 in the category of Environment and Climate Change, and all but one for the categories of Urban Planning (Utqiaġvik) and Governance (Reykjavik). Whitehorse and Reykjavik either underperformed in comparison to ISO 37120 or excluded altogether several thematic areas, including Education, Finance, Health, Housing, Safety, and Telecommunication.

**Table 5:**
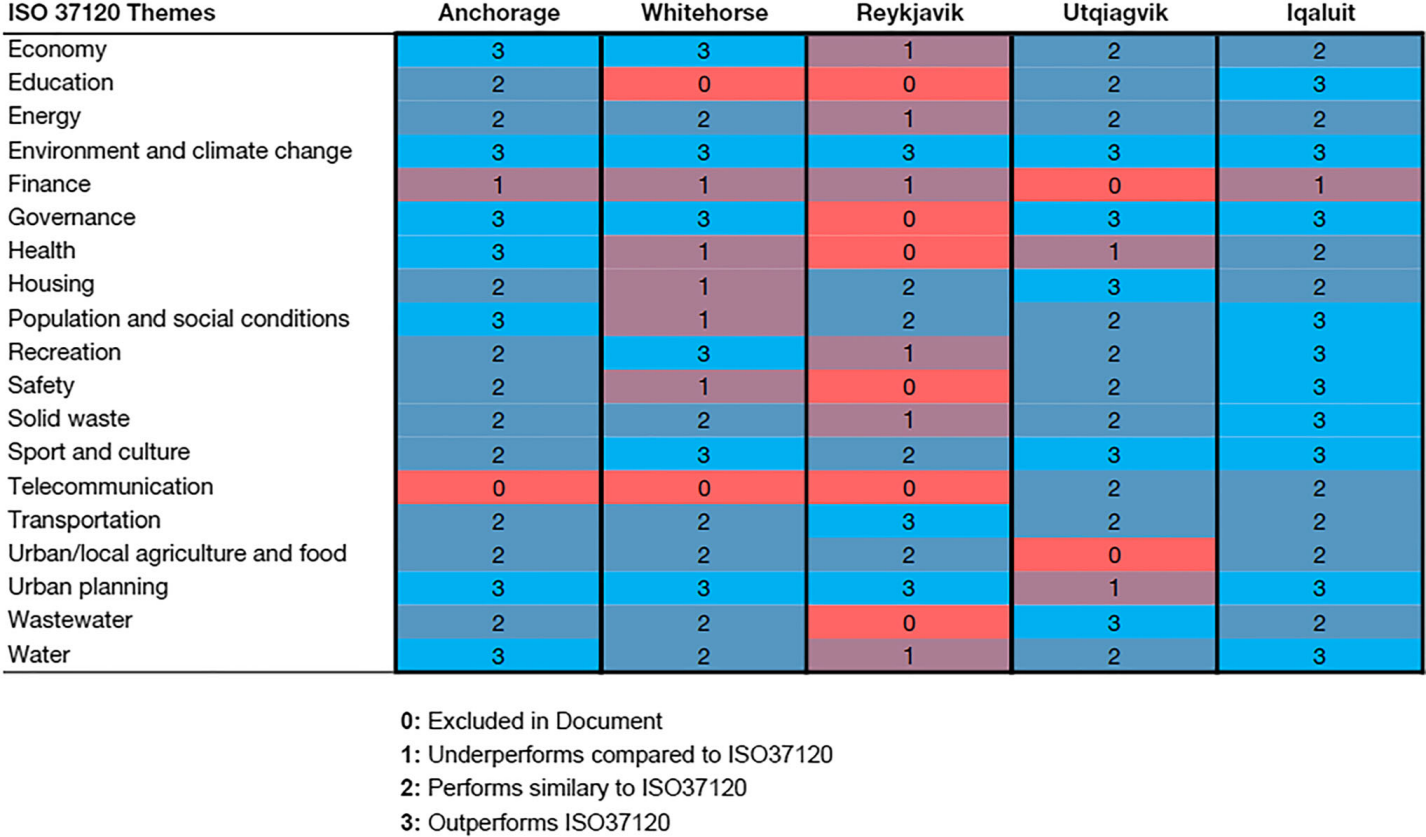
Comparison of ISO 37120 to the planning frameworks for five Arctic cities

The average scores for each thematic area for the five cities are shown in Table 6. Across the reviewed plan frameworks, eight areas underperformed in comparison to ISO 37120, ranging from Energy (average score: 1.8) to Health (1.4), with Telecommunication (0.8) and Finance (0.8) scoring lowest across all cities. Only two thematic areas performed similarly in comparison to ISO 37120: Housing (2) and Solid Waste (2). The remaining nine areas all outperformed ISO 38120, including Sport and Culture (2.6), Urban Planning (2.6), Governance (2.4), with Environment and Climate Change (3) scoring highest among all cities.

**Table 6:** Summary of average combined score by thematic area in comparison to ISO 37120

ISO 37120 themes	Avg. score of evaluated frameworks	Rank	Notes
Environment and climate change	3	1	More comprehensive than ISO 37120
Sport and culture	2.6	T-2	
Urban planning	2.6	T-2	
Governance	2.4	4	
Economy	2.2	T-5	
Population and social conditions	2.2	T-5	
Recreation	2.2	T-5	
Transportation	2.2	T-5	
Water	2.2	T-5	
Housing	2	T-10	As comprehensive as ISO 37120
Solid waste	2	T-10	
Energy	1.8	T-12	Less comprehensive than ISO 37120
Wastewater	1.8	T-12	
Safety	1.6	T-14	
Urban/local agriculture and food security	1.6	T-14	
Education	1.4	T-16	
Health	1.4	T-16	
Finance	0.8	T-18	
Telecommunication	0.8	T-18	

Table 7 ranks each city included in this study by their average performance across all 19 ISO 37120 categories of indicators. Although having the smallest population of the 5 cities, Iqaluit scored highest, presenting the most comprehensive sets of goals, targets, and indicators, and outperformed Anchorage, the largest city included in this study. On average, Utqiaġvik and Whitehorse performed similarly with respect to the type of indicators in their planning frameworks as ISO 37120, scoring near 2. Reykjavik underperformed across multiple thematic areas and ranked lowest overall in terms of useful metrics to measure sustainability when compared to ISO 37120.

**Table 7:** Summary of average combined score for each city in comparison to ISO 37120

City	Overall average score	Rank	Notes
Iqaluit (CA)	2.5	1	Cities with avg. scores > 2 tend to be outperform ISO 37120. cities with avg. score < 2 tend underperform in comparison to ISO 37120
Anchorage (USA)	2.2	2	
Utqiagvik (USA)	1.9	3	
Whitehorse (CA)	1.8	4	
Reykjavik (IS)	1.2	5	
Overall average score	1.9		

## Discussion

According to Vlasova et al. (2020), Arctic sustainable development practice and policy should consider not only the “health, wellbeing, and security of Arctic communities,” but also engage more directly with issues of ecology, equity, and knowledge co-production, including those inclusive of Indigenous populations. The analysis presented here shows the degree to which approaches to planning differ among the five Arctic cities considered in this study. While such differences do not necessarily indicate deficiencies in the utility, intent, clarity, or effectiveness of a city’s plans for sustainable development, they do reflect a range of priorities for each city that result from issues of scale, context, and capacity. For example, Reykjavik, a highly urban city with European planning influences in a relatively temperate climate, defines 55 goals across four broad themes that encompass many areas of urban life, with a strong emphasis on urban development and growth (Reynarsson 1999). Iqaluit, a small, remote, and cold city with a majority indigenous population also organizes its long-term visions across four themes (and 59 goals) but prioritizes community planning and sociocultural outcomes. Utqiaġvik, lacking a vision plan, organizes its primary goals by sector of city services, covering the least number of goals of all cities (21), but that results in twice as many sets of subthemes as the other four cities (182). Whitehorse uses different sets of organizing themes between its vision and comprehensive plans but is unique in that the city defines an additional group of broad principles in its comprehensive plan that are used to organize more detailed objectives and policies. Anchorage defines its goals by sector of city services in its vision plan, while its comprehensive plan focuses almost entirely on the technical applications of land use policy as the driving implementation tool to guide growth in the city.

These characteristics of urban sustainability planning for each of the five cities are important and affirm that Arctic cities, across a wide variety of strategies and methods, seek to provide services to their residents and surrounding communities like cities in other regions of the world. Our analysis reveals that, for the cities in this study, ISO 37120 provides sufficient or greater metrics to monitor sustainability performance across just over half of its thematic areas. For certain categories, such Education, Health, Finance, and Telecommunication, the five cities consistently underperformed in comparison to ISO 37120 or excluded both indicators and goals in those areas. The potential explanations for this lack of coverage are twofold: either cities simply do not provide sufficient detail in their planning documents to meet the standards or metrics set by ISO 37120, or that these themes do not reflect essential areas or priorities for the provision of city services. Significantly, however, the five cities outperform ISO 37120 across a total of nine of 19 thematic areas, of which the highest scoring thematic areas include Environment and Climate Change, Culture, Urban Planning, and Governance. From these results, it is possible to evaluate the specific differences between both sets of indicators to develop a better understanding of the approaches taken by Arctic cities to plan for and monitor progress toward achieving greater sustainability.

For example, in Environment and Climate Change, ISO 37120 only provides core indicators for greenhouse gas emissions, species diversity, and the conservation of natural areas, whereas the frameworks reviewed in this study present metrics that describe land and natural resource management, human-wildlife interactions, and natural hazards (e.g., flooding, erosion, subsidence, and permafrost thaw). The plan frameworks also present goals, targets, and indicators that consider the impact of climate change on these environmental areas. For example, both Anchorage and Utqiaġvik establish strategies and indicators that reflect (1) health and safety issues resulting from climate impacts, (2) the development and protection of community infrastructure, (3) public access to climate change action planning resources, and (4) coordination of activities between stakeholders on projects and activities related to the natural environment.

ISO 37120 provides only one core indicator in Sport and Culture—the number of cultural institutions and sport facilities per population, and two supporting indicators—the percentage of municipal budget allocated to those facilities and the annual number of cultural events. Many of the plans reviewed in this study provide additional metrics that evaluate heritage management—efforts to protect unique social and cultural identities of Arctic cities, including those involving traditional or indigenous resources and activities. For example, Utqiaġvik provides 13 indicators ranging from the preservation of traditional hunting, fishing, and gathering areas to the protection of historic built structures. Iqaluit also provides specific metrics for cultural education and accessibility and communication of city services to indigenous populations, such as the number of programs offered in the Inuktitut language for municipal staff or the number of public spaces that contain Inuit art or cultural representations.

In Governance, the cities in this study demonstrated through indicators, goals, and targets the joint participation of communities and stakeholders in local governance, including the number of community-led initiatives or projects resulting from city–stakeholder partnerships to the percentage of population that regularly volunteers. Cities with large indigenous populations, such as Utqiaġvik and Iqaluit, also track the roles of tribal participation in local planning and governance. In contrast, ISO 37120 only covers issues of voter participation and diversity and equality among city officials across a total of four indicators.

Lastly, ISO 37120 provides general and descriptive metrics in Urban Planning, such as population density, green area per population, proximity to basic services, and jobs-housing ratio, among others. The frameworks reviewed in this study present more specific indicators and goals that provide targeted measures of the impacts and results of urban development and growth, such as, mixed-use and residential density in downtown cores, diversity of new businesses in downtown cores, hazard mitigation strategies in new developments, built-up density in historic preservation areas, and built-up density per district or neighborhood. Certain cities, such as Reykjavik, also presented indicators that reflect public participation in planning processes, such as the number of open (public) planning and design competitions. Notably, larger cities tend to apply ISO 37120 indicators in multiple ways across the various districts of the city —for example, population density or green area by neighborhood, which are linked to the corresponding subdivision of land uses across the city.

Overall, for Arctic cities that are increasingly vulnerable to the impacts of climate change, evolving policy practices, and local-based economies, it is important for cities to both establish goals for future growth as well as implement tools to measure and monitor progress toward achieving those objectives. Sustainability indicators are critical to this approach by revealing the direction of progress across various sectors of city services and functions over time. However, the use of indicators also presents challenges in terms of reliability, specificity, repeatability, and completeness. The research presented in this paper reveals the shortfalls of applying a standardized international SDI framework such as ISO 37120 to Arctic cities. This effort also reveals the ways in which cities structure their sustainability plans in relation to broader efforts to develop long-term sustainability goals. The set of documents and analysis presented in this paper is not exhaustive, as data vary depending on public accessibility to city planning documents, as well as differences in policy requirements and responsibilities at the municipal, regional, or national levels to prepare and publish sustainability plans. This set of plans also does not include a review of secondary planning documents that supplement or inform primary planning documents used by each city for the purposes of sustainability and land use planning.

One potential downside of the type of analysis conducted is that a low score (i.e., instances where a city underperforms in a certain thematic area in comparison to ISO 37120) does not definitively mean that area is not an important or considered area of sustainability planning for that city. The results of this analysis only indicate where a city does or does not provide a tool that measures the results of or progress toward a goal, target, or objective that meets the standards set by ISO 37120. It is possible that such metrics may be provided by secondary planning documents not reviewed in this study. Thematic areas that were excluded in the reviewed plans could also be the responsibility of another municipal department or of another authority at a higher administrative level (e.g., a regional plan) that may not have a direct relationship to the planning work being done by the city. Further review would be required to expand to additional secondary and unofficial planning documents that are beyond the scope of this study.

Each of the five cities approaches sustainability planning in a different way, forming a gradient of approaches that result from the type of city and its specific geographic, economic, and sociocultural context. Some are thematic and organized into high-level visions (Reykjavik's “A City for People”) or principle-driven statements (Whitehorse’s “Strong Downtown and Livable Neighborhoods”) that cover multiple areas of sustainable development. Other frameworks are technical, such as Anchorage and Utqiaġvik, emphasizing line-item sectors of city services. This variation reflects differences in priorities, strategies for communicating sustainability planning, and the diversity of long-term goals for each city. A significant result of this study is that ISO 37120 is less suited to measure progress toward achieving sustainability for those cities that describe broad visions organized across fewer themes. For example, Reykjavik presents 55 goals over four primary themes: City by the Sea (urban development), The Creative City (business development), The Green City (ecological districts), and A City for People (urban quality); whereas Anchorage presents 35 goals over 10 themes (three times less broadly organized). Both cities have robust planning frameworks, but Reykjavik scored the lowest among all cities in comparison to ISO 37120 (1.2), underperforming across 12 of 19 categories.

It is also important to make evident that Iqaluit and Utqiaġvik are Indigenous-governed cities. While challenges persist today regarding the weight of Indigenous leadership within larger political and economic administrations, both cities present unique and different relationships to social and cultural outcomes that result from sustainability planning than in other cities, both in this study and throughout the Arctic region (Zanotti et al. 2020; Gladstone and Dalseg 2022). Iqaluit, established as the capital of Nunavut in 1999 following the Nunavut Land Claims Agreement (1995), has the highest population of Inuit of all Canadian cities (over half). Utqiaġvik is the largest Inupiaq community of the North Slope region, where over 60 percent of the community identifies as Alaska Native, and is a major regional hub as the seat of the home-rule North Slope Borough government. Through self-rule and the participation of local leaders in governance, more appropriate outcomes can be generated for the populations of the cities, including “place sensitive design, diversity, sustainability, and promotion of social cohesion” (Petrov et al. 2013). Such focus on Indigenous values also allows for local livelihoods to help re-shape discourses, practices, and strategies for collective wellbeing, support decision making, set goals for outcomes relevant to local communities and other stakeholders involved in local development, and maximize the benefits of projects or policies established through city planning.

This analysis also reveals certain issues with the ability of ISO 37120 to reflect either the capacity or results of local planning efforts. First, ISO 37120 does not consider metrics that reflect the various challenges to implementing policy defined in sustainability plans and specific to Arctic cities outside of capital expenditures. For example, the cities in this study provided indicators to monitor the extent, condition, or availability of urban infrastructure, permafrost, paved roadways, or gravel resources for new construction; waste management infrastructure; and the ratios of public versus private financing for new or ongoing capital projects, among others. Such factors have large impacts on the ability for a city to implement planning policy (i.e., deliver projects)—resulting from increased cost cycles, longer timelines, seasonality, and multistakeholder coordination—thus impacting the overall sustainability profile of a city. Second, ISO 37120 does not include indicators that evaluate the types, quality, or contributions of projects resulting from planning policy as they relate to different areas of sustainable development (e.g., the number of adaptive re-use projects, or the number of projects that include recreational or outdoor public spaces). And third, both ISO 37120 and the cities reviewed in this study do not include indicators that evaluate the extent to which a city considers sustainable development a priority, for example: *Is there a planning office in the city? How many sustainable development plans has the city published? How many plans are publicly available? What is the lifespan and review period of the sustainability plan?*

Notably, SDI frameworks such as ISO 37120 can extend significant benefits by allowing for shared approaches to urban sustainability planning among diverse groups of cities at multiple scales of governance, while also permitting comparative insight and global benchmarking (McCarney 2015). An SDI framework that reflects more local-level issues relevant to Arctic cities can lead to a greater coordination of responses in terms of sustainability planning, which can in turn lead to an increase in urban resilience. However, the inclusion of local-level issues that reflect specific contexts, like those described above, can conversely make an SDI framework less applicable among larger groupings of cities and more difficult to share the results. While this paper has identified the importance of certain thematic areas and their corresponding indicators for the Arctic cities included in this study, these indicators are not consistent from city to city due to varying contexts. Further research is required to define the set of indicators that reflect shared sustainable development priorities among all cities with a greater emphasis on indicators that can reflect the results of the implementation of such policy on the city itself.

## Conclusion

Standardized international sustainability frameworks (indexes) such as ISO 37120 are often used to compare data across cities globally using comprehensive sets of urban sustainability indicators. Such indexes can help form a complete picture of the sustainability of a city by measuring progress toward urban, socioeconomic, and environmental goals established by planning authorities. However, while comprehensive in terms of thematic areas covered, they lack relevance to any specific context and have difficulty characterizing future visions of the city in terms of sustainable development. The research presented in this paper evaluates the ability of ISO 37120 to adequately characterize sustainable development planning goals and outcomes that are specific to cities located in the Arctic region through a detailed review and analysis of a subset of Arctic cities’ plans for sustainable development and their corresponding organizations, goals, targets, objectives, and indicators. This paper also provides a discussion on the differences in approaches to sustainability planning in Arctic cities and the ways in which cities structure their plans in relation to broader efforts to develop long-term sustainability goals. The initial review of the cities’ planning documents revealed that all cities have a comprehensive plan that is the primary legally binding document enabling a city to carry out policies for future growth, but there was not a significant relationship between this and whether cities prepare or publish additional planning documents such as sustainability, climate, or land use documents.

When a subset of these cities’ plans and their corresponding thematic frameworks were compared directly to ISO 37120, it was found that Iqaluit, Anchorage, and Utqiaġvik have more comprehensive planning frameworks than Whitehorse and Reykjavik, with almost all the thematic areas either meeting or exceeding metrics presented by ISO 37120. Eight thematic areas underperformed in comparison to ISO 37120, including Energy (average score: 1.8), Health (1.4), Telecommunication (0.8), and Finance (0.8). Often these areas were not covered topically by the cities’ plans or did not include any indicators. The highest scoring categories included Environment and Climate Change (3.0), Sport and Culture (2.6), Urban Planning (2.6), and Governance (2.4), which reflect the widely accepted pillars of sustainable development: environmental management, social and economic development, and urban governance. In these categories, cities included addition indicators that measure and monitor the results of (1) land and natural resource management, human–wildlife interactions, and natural hazards, (2) heritage management and preservation of cultural identity, joint and public participation in local governance and planning, including tribal participation, and (3) the impacts of urban development and growth per district, zone, or neighborhood. A significant result of this study revealed that ISO 37120 is less suited to measure progress toward sustainability for those cities that, in their planning documents, describe broad visions organized across fewer themes and is more suited to reflect plans structured around sectors of city services. Overall, such differences described above reflect the range of priorities for each city that result from issues of scale, context, and capacity. These include more relevant, cohesive, and place-sensitive outcomes that result from both self-rule and the participation of indigenous peoples in local governance. The results of this research further indicate that ISO 37120 works to benchmark sustainability for universal attributes of cities but by lacking locally specific indicators that address the unique concerns of individual communities fails to address areas where Arctic cities are unique. While SDI frameworks are important tools that allow cities to track progress toward achieving sustainable futures, further study is needed to identify and incorporate indicators that provide greater context, cross scales, and better reflect the goals and impacts of projects or development that result from sustainability planning.

### Supplementary Information

Below is the link to the electronic supplementary material.

Supplementary file 1 (PDF 150 KB)

## Footnotes

1 Arctic Human Development Reports.

2 Arctic Monitoring and Assessment Program.

3 Conservation of Arctic Flora and Fauna Working Group.

4 Emergency, Prevention, Preparedness and Response Working Group.

5 The term “vision plan” was used by the cities’ plan documents reviewed in this study and can also refer to other non-binding plans such as sustainability plans, community plans, or action plans, among others.
