# Supplementary material for: Evaluating plans for sustainable development in Arctic cities
Source: Ambio. 2024 Apr 9;53(8):1109–23. doi: 10.1007/s13280-023-01974-6 (PMC11183006; doi:10.1007/s13280-023-01974-6)
Supplement: Supplementary file 1 — (PDF 150 KB) [file 13280_2023_1974_MOESM1_ESM.pdf]

Ambio

Electronic Supplementary Material

Title: **Evaluating plans for sustainable development in Arctic cities**

Authors: Benjamin DiNapoli, Matthew Jull

Table S1 Urban profiles of 5 Arctic cities used for detailed comparative analysis.

Context	City	Anchorage	Utqiagvik (Barrow)	Iqaluit	Whitehorse	Reykjavík
	Place	Alaska, USA	Alaska, USA	Nunavut, CA	Yukon, CA	Iceland
Urban Characteristics	Settlement Date	1914	1959	1942	1920	1786
	Population	291,247	4,927	7,429	28,201	141,010
	Latitude	61.2175°	71.2906°	63.7467°	60.7209°	64.1355°
	Mean Jan./July temp. (C°)	-7.5 (14.9)	-24.4 (5)	-24.7 (7.8)	-16.4 (14.2)	0 (11.4)
	City area (km²)	257.1	55.6	52.5	38.2	95.3
	Significance	Major international air and sea freight hub	Scientific hub for Arctic, climate change, and military research	Former northern military defense station; largest Inuit populations in Canada	Largest city in the Yukon Territory for mining, energy and wilderness tourism.	Major international air transportation center
	Primary Industries	Resource extraction; shipping; tourism	Resource extraction	Resource extraction; tourism; subsistence	Energy; resource extraction; government	Tourism; manufacturing; fisheries
	Indigenous Peoples	Yup'ik	Inupiat	Inuit	First Nation	N/A
